# Optical Three-Dimensional Imaging for Objective Evaluation of the Donor Site after Anterolateral Thigh Flap Surgery

**DOI:** 10.3390/jcm13061805

**Published:** 2024-03-21

**Authors:** Marius Heitzer, Philipp Winnand, Mark Ooms, Anna Bock, Marie Sophie Katz, Florian Peters, Kristian Kniha, Stephan Christian Möhlhenrich, Frank Hölzle, Ali Modabber

**Affiliations:** 1 Department of Oral and Cranio-Maxillofacial Surgery, University Hospital RWTH Aachen, Pauwelstraße 30, 52074 Aachen, Germany; 2 Department of Orthodontics, University of Witten/Herdecke, Alfred-Herrhausen Str. 45, 58455 Witten, Germany

**Keywords:** transplant donor site, three-dimensional imaging, free flaps, wound healing diagnostic imaging

## Abstract

**Background:** The high volume of the fasciomyocutaneous anterolateral thigh flaps (ALT) is suitable for the reconstruction of pronounced soft tissue defects. At the same time, harvesting ALT results in a drastic change in thigh shape. Here, we present an optical three-dimensional imaging method for thigh comparison, which can be an objective and reproducible method for evaluating donor sites after ALT harvesting. **Methods:** In total, 128 thighs were scanned with an optical three-dimensional scanner, Vectra XT ^®^. Sixty-eight non-operated right and left thighs were compared and served as a control. Sixty thighs were scanned in the ALT group. The average surface area deviations, thigh volume, thigh circumference, and flap ratio to thigh circumference were calculated. The results were correlated with Δthigh circumference and Δvolume of the unoperated thighs of the control group. **Results:** No significant difference between the thigh volumes of the right and left thighs was found in the control group. Removal of an ALT flap showed a significant (*p* < 0.007) volume reduction compared to unoperated thighs (2.7 ± 0.8 L and 3.3 ± 0.9 L, respectively). Flap area correlated strongly with the Δthigh circumference (r = 0.66, *p* < 0.001) and Δvolume (r = 0.68, *p* < 0.001). Strong correlations were observed between flap ratio and thigh circumference with Δhigh circumference (r = 0.57, *p* < 0.001) and Δvolume (r = 0.46, *p* < 0.05). **Conclusions:** Optical three-dimensional imaging provides an objective and reproducible tool for detecting changes in thigh morphology volume differences after ALT harvesting.

## 1. Background

The free anterolateral thigh flap (ALT) is an integral part of reconstructive surgery due to its versatility in tissue replacement of reconstructions of tissue defects, especially of the head and neck [1,2,3,4,5]. Its versatility of use is based on the possibility of elevating this flap as a cutaneous, fasciocutaneous, or myocutaneous flap [2,6]. Furthermore, the ALT flap, in addition to its adequate pedicle length [7,8], offers a versatile flap design [2] and safe flap elevation [6], which, in combination, provide ideal characteristics for the reconstruction of large and complex soft tissue defects [8,9]. Since ALT flaps are used particularly for the reconstruction of large tissue defects, a corresponding volume deficit results in the area of the donor site [10]. Previous studies mainly focused on the impact on the recipient region according to the vascular anatomy [7,11,12], the volume [1,2], the flap size [1,2], and clinical applications of ALT flaps [2,3,8]. Considerably less frequently, studies have examined morbidity at the harvest site [6,13,14,15,16], for example, in the form of gait abnormalities or lower extremity weakness [9,17,18] after free ALT fasciomyocutaneous flap harvest. To date, few studies have examined healing at the donor site and evaluated wound healing over a prolonged postoperative period [6]. Moreover, the literature currently lacks valuable data on the assessment of femoral volume changes in the postoperative course after wound healing following ALT flap surgery.

There is a vibrant search for an objective, cost-effective, rapid, valid, and noninvasive method to evaluate the leg volume of the ALT donor site. Current methods for the standardized quantification of legs and lower limbs include questionnaires [10,13,18], tape measurements [18,19,20], water displacement [21], perometire [19], and computer tomography (CT) or magnetic resonance imaging (MRI) [22]. Limitations of these methods include unreliable estimation of band measurements [19] and the cumbersomeness of water displacement, which is particularly inappropriate for patients with open skin lesions [23]. Radiological imaging is used to evaluate structural changes after interventions [24]. MRI and CT imaging, on one hand, offers the advantage of allowing valid volume determination of body structures by segmentation of cross-sectional images, which, on the other hand, are time-consuming to implement and, in the case of CT imaging, fraught with radiation exposure, making these methods unsuitable for the routine monitoring of morphological structural changes [25]. Despite the aforementioned limitations, circumference measurement with a tape measure is still frequently used, as this method does not require sophisticated technology and is inexpensive.

Three-dimensional imaging with modern three-dimensional scanners is becoming increasingly popular in medicine and represents a valuable tool for the objective documentation of volumetric body morphologies [26,27,28,29,30,31]. According to Kroh et al., this is partly because these scanners do not use harmful ionizing radiation and are easy to use with mobile and modern terminals [26]. In addition, detailed three-dimensional imaging is easily reproducible and enables objective data acquisition and post-processing of the image information [26,27]. Consequently, these three-dimensional data have the potential to be part of complex surgical planning and are also used for postoperative outcome assessment in reconstructive and plastic surgery [26,29,31,32]. In addition, Etzel et al. described reproducible and accurate digital volume determination of the legs using acquisition by three-dimensional whole-body surface imaging, which provides the ability to monitor morphologic changes in multiple areas without the need to individually scan each anatomic region of interest [25]. The desktop-based Vectra XT (Canfield Scientific, Inc., Parsippany, NJ, USA) is a well-established modern whole-body surface scanner, which has already demonstrated high accuracy and measurement precision of the acquired three-dimensional data in clinical studies [33,34].

The aim of the present study is to investigate the feasibility of a three-dimensional assessment of thigh morphology and donor site evaluation in patients after ALT flap surgery compared with the volume of the unoperated thigh. A preliminary study of non-operated patients to verify the comparability of both thighs in one individual was performed and transferred to patients with ALT. These generated three-dimensional data were compared and correlated with conventionally clinically obtained data from patients with ALT.

## 2. Methods

All methods were carried out in accordance with relevant guidelines and regulations of ethical approval by the Ethics Committee of the Medical Faculty of RWTH Aachen University (EK 23-077). Written informed consent for photographic documentation was obtained from all patients, and 128 thighs from 64 patients were retrospectively studied. All patients underwent microsurgical transplantation and were evaluated 22 ± 15 months postoperatively. The control group consisted of 34 patients (age 66 ± 11 years), 16 females (age 62 ± 21 years) and 18 males (age 66 ± 12 years), all of whom had a history of malignant tumor disease that was treated by other transplants not derived from the lower extremities. The ALT group consisted of 30 patients (age 70 ± 19 years), 12 females (age 69 ± 26 years) and 18 males (mean age 64 ± 14 years), who had had a fasciomyocutaneous ALT flap taken from one of the thighs (Table 1).

All operative procedures were oncologic maxillofacial resections with the direct reconstruction of patients with squamous cell carcinoma (*n* = 25), sarcoma (*n* = 3), mucoepidermoid carcinoma (*n* = 1), and sweat gland carcinoma. A total of 26 ALT flaps were harvested from the right thigh, and 4 ALT flaps were harvested from the left thigh. All ALT flaps were faciomyocutaneus ALT flaps, and donor sides were closed primarily without a skin graft necessary for closure. The age, sex, body mass index (BMI), and femoral circumference were obtained from the clinic documentation. The area of the anterolateral thigh flap in centimeters was obtained from the surgical records, and the flap ratio to thigh circumference (in percent) was determined. Exclusion criteria were no written consent for photographic documentation in the patient record, postoperative duration of fewer than 6 months, harvest of a bilateral ALT flap, and previous surgery or orthopedic disease of the legs.

### 2.1. Circumference of Thighs

According to an established protocol for the evaluation of thighs in the context of ALT operation, thigh circumference was measured on both thighs at the midpoint of the junction line between the lateral superior border of the patella and the anterior superior iliac spine [20]. All measurements were taken by an experienced surgeon using the same spring-loaded tape measure. The flap width was obtained from the operation record. Calculation of the flap ratio to thigh circumference was conducted according to an established protocol [35]. 

### 2.2. Three-Dimensional Imaging and Analysis

Three-dimensional scanning was performed using the Vectra XT 3D imaging system (Canfield Imaging Systems, Fairfield, NJ, USA), which is used for standard care in our hospital. The six color cameras of this floor-standing scanner are positioned in a triangular configuration and can capture 180° images. To ensure high scan quality, subjects were asked to remove all jewelry and pants, with the exception of tight-fitting underwear. Clothes covering the upper body were rolled up to the navel. The camera system is adjustable to body height. Imaging was performed from the front in a standing position according to an established protocol at a scan distance of 1 m, with foot markers on a metal stool [25]. The patients stood upright with eyes opened and arms folded behind the back. The generated images were processed into a high-resolution three-dimensional image model using integrated software. Subsequently, the obtained data were exported into OBJ file format for further analysis. According to references in the literature, data were processed using Geomagic Control 2014 software (3D Systems Corporation, Rock Hill, SC, USA) [26]. To allow comparability of the thighs, the 180° ventral imaged thigh portions of the image file were cropped along the junctional line between the anterior superior iliac spine and the inguinal fold at the top and by a horizontal tangent on the superior edge of the patella at the bottom using the integrated snipping tool of the software. The cropped file was then doubled. To achieve an exact fit of the legs, the left leg was removed with the snipping tool, and the remaining right leg was doubled and mirrored along the vertical alignment. Thus, one file has a right thigh and a mirrored right thigh. The other file has a regular left thigh and a regular right thigh. The superimposition of the two exact right thighs allowed for the perfect fit of both files. The overlay of the two files was created using the software’s built-in best-fit algorithm. Subsequently, to the best fit, the maximum deviation between the legs was determined using Geomagic Control software. For the patients after ALT surgery, the leg with the donor side served as a unique landmark as well as a reference, and this thigh was also mirrored along the vertical alignment, as described above. The software-based best-fit overlay and the determination of the maximum deviation were performed in the same way as for the control group. The volume of the thighs trimmed on the computer program was defined individually for each thigh by a bounding plane in the frontal layer at the posterior border of the optical surface image, and the volume specification was determined using Geomagic Control 2014 software.

### 2.3. Statistical Analysis 

Graph Pad Prism for Mac (version 9.0, GraphPad Software, La Jolla, CA, USA) was used to perform statistical analyses and generate graphical representations of the data. The results are presented as the mean ± standard deviation unless otherwise specified. The normal distribution of the variables was checked using the D’Agostino and Pearson normality tests. Appropriate normal distributed data were then analyzed, and the significance of the differences between the groups was determined using the unpaired Student’s *t*-test. The data for maximum three-dimensional deviation were not normally distributed and analyzed using the Mann–Whitney U test. The correlations between Δvolume, Δthigh circumference, flap ratio to thigh circumference, and flap area were evaluated using the Pearson correlation test. Differences were considered significant when *p* < 0.05.

## 3. Results

The patient characteristics are shown in Table 1. The mean body weight of the patients in the control group was 76 ± 21 kg, with a mean BMI of 25 ± 4.7 kg/m^2^. Patients with ALT surgery had a mean body weight of 69 ± 16 kg and a mean BMI of 23 ± 4.4 kg/m^2^. The average flap area of the lifted skin island was 73 ± 41 cm^2^.

Postoperatively, the thigh circumference was 44 ± 3 cm on the non-operated side, and a significantly lower mean circumference of 40 ± 2.3 cm was measured on the donor side (*p* ≤ 0.001), leading to a Δthigh circumference of 3.9 ± 2.4 cm. The calculated flap ratio to thigh circumference between the operated and unoperated thighs of the ALT group was 15 ± 4.6% (Table 2).

**Figure 1:**
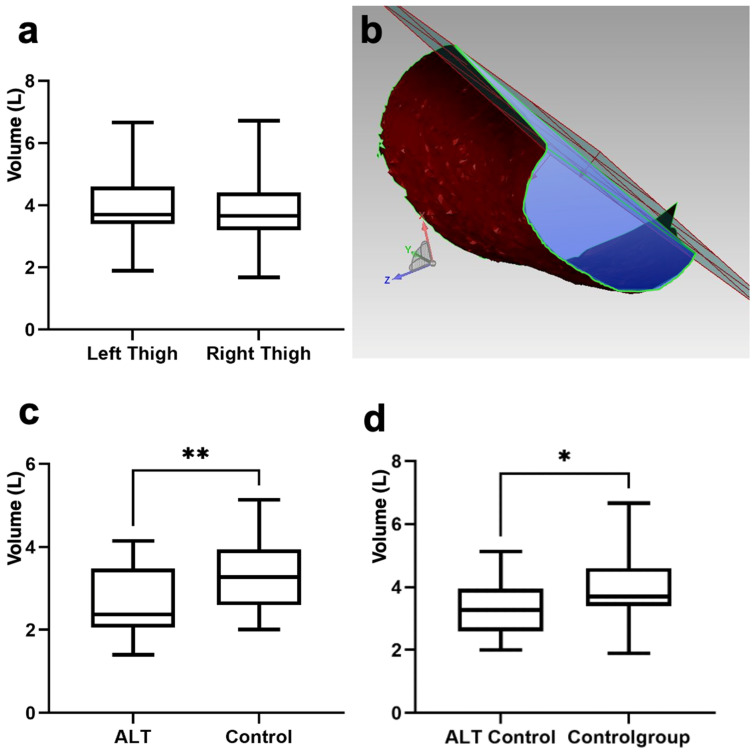
(**a**) Thigh volume of the control cohort; (**b**) image of three-dimensional computer-based volume determination; (**c**) thigh volume of the ALT cohort and control cohort; (**d**) thigh volume of the control leg of the ALT cohort (the non-operated leg) and thigh volume of control cohort. ** *p* < 0.01; * *p* < 0.05.

In the control group, there was no significant difference in the thigh volumes of both legs between the right thigh (3.7 ± 1 L) and the left thigh (3.8 ± 0.9 L). However, the thighs from which an ALT flap was taken showed a significantly lower volume, with a mean volume of 2.7 ± 0.8 L, compared to the volume of the non-operated thighs, with 3.3 ± 0.9 L (*p* < 0.01). The Δvolume of thighs of the control group was 0.2 ± 0.2 L, and the Δvolume of the ALT group was 0.6 ± 0.4 L (Table 3 and Figure 1).

**Figure 2:**
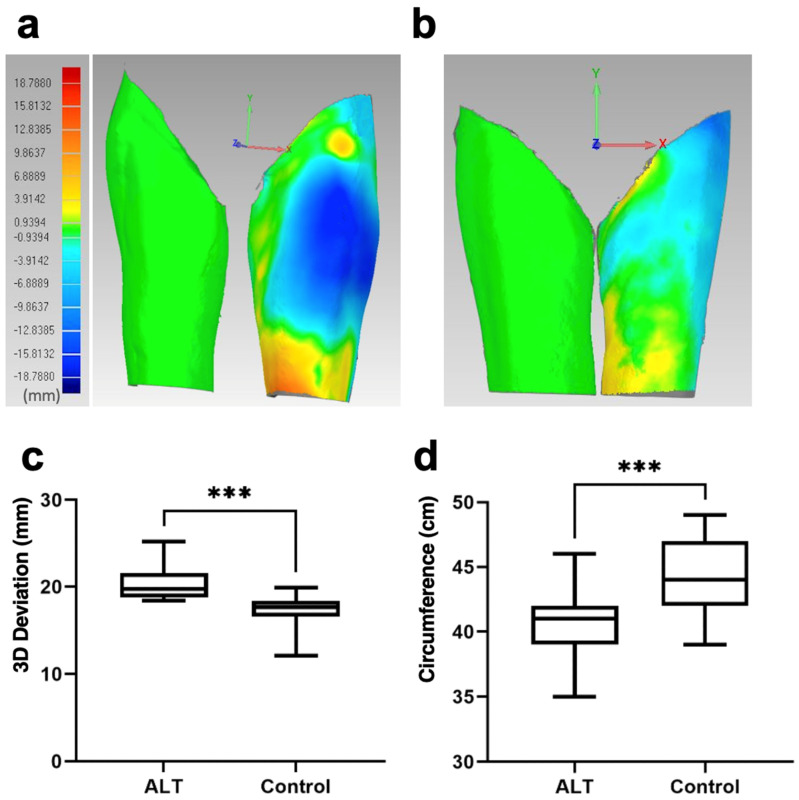
Thermal maps of the superimposed matched scans of the thighs with joined mirrored thighs. (**a**) ALT group donor site (right patient side) and thermal map of superimposed mirrored donor site with the unoperated thigh (left patient side with negative values). (**b**) Unoperated legs of the control group with nearly no difference in the thermal map of superimposition; (**c**) deviations of matched thighs of ALT cohort; (**d**) differences in circumference of thighs of ALT cohort. *** *p* < 0.001.

Thermal maps of the two superimposed matched scans of the thighs illustrate the areas where the mirrored thighs were joined without spacing by the software-based best-fit tool, and the other thigh shows the divergence of thigh shape between the two thighs and in the ALT group between the donor site and the unoperated leg of the opposite side (Figure 2a,b). In the control group, the maximum three-dimensional deviation between the right and left thighs was 17 ± 1.9 mm (Figure 2c). In contrast, in the ALT group, the mean maximum three-dimensional deviation of 20 ± 2.1 mm (*p* < 0.001) was significantly higher (Figure 2d).

**Figure 3:**
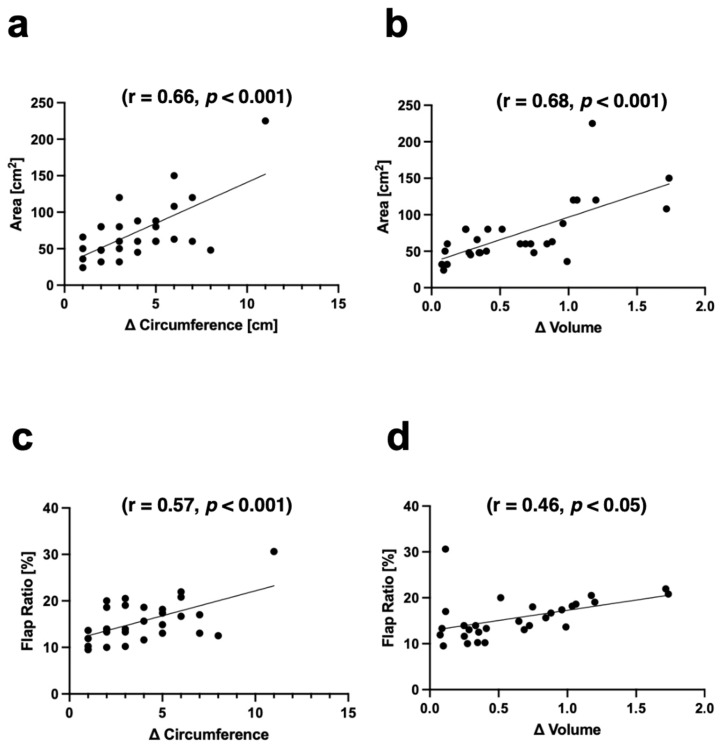
Scatter plots of (**a**) flap area versus Δthigh circumference; (**b**) flap area versus Δthigh Volume; (**c**) flap ratio versus Δthigh circumference; (**d**) flap ratio versus Δthigh volume.

A significant positive correlation was found between flap area and Δthigh circumference (r = 0.66, *p* < 0.001) as well as between flap area and Δvolume (r = 0.68, *p* < 0.001). A significant positive correlation was also found between flap ratio and thigh circumference, with Δthigh circumference (r = 0.57, *p* < 0.001) and Δvolume (r = 0.46, *p* < 0.05) (Figure 3).

## 4. Discussion

According to the introduction of Song et al. in 1984 [36], the ALT flap has gained popularity in the last decade [3,4,5,6,8,14]. The reliability and high success rates of ALT flaps have allowed an increased focus on the planning [1] and appearance of the recipient region [2], but now, the evaluation of the donor sites is also increasingly part of investigations [13,14,16]. The operation of a fasciomyocutaneus or faciocutaneus ALT flap affects the donor site to varying degrees, which is why only fasciomyocutaneus ALT flaps were examined. In this study, a novel evaluation of thigh measurement and thigh volume after the harvest of an ALT flap from three-dimensional surface imaging was described and evaluated. The data processing method was specifically designed to be valid and reproducible.

Thigh circumference and BMI are often directly related, and this must be considered when examining thighs in a patient population. In our study, the BMI of the patients was 23 kg/m^2^. In a multi-institutional review study, Turin et al. described an analogous mean BMI of 25 kg/m^2^ of patients with ALT flap operation [14]. Weise et al. described the differences in thigh circumference measurements, which were 15 cm above the knee joint space, with a reduction of 99% on the donor side after harvest of ALT flaps [18]. In this study, circumference measurements for both legs revealed that the donor side was reduced to 91%. This difference can be explained by the following two circumstances: first, the measurement points were located in different parts of the thighs, and second, the mean flap area of harvested ALT flaps in our study included a slightly larger area of 73 cm^2^ compared to 70 cm^2^ [18]. In our study, significant positive correlations were found between flap area and Δthigh circumference (r = 0.66, *p* < 0.001) and between flap ratio to thigh circumference with Δthigh circumference (r = 0.57, *p* < 0.001). Nevertheless, the measurement method used in this study has significant advantages. The use of an individual but standardized measurement point, which is located at the midpoint of the junction line of the lateral superior border of the patella and the anterior superior iliac spine, allows for the accurate and reproducible measurement of the thigh diameter at the exact midpoint of the thigh. Furthermore, the different leg lengths of the patients were considered using this measurement method. Investigations by Boca et al. [20] reported preoperative thigh circumferences of 48 cm using the same measurement method and are consistent with the determined data from the unoperated thighs with a diameter of 44 cm in this study.

Another established method for evaluating thighs in the context of ALT flaps described in the literature is the flap ratio to thigh circumference values [15,20]. The flap ratio to thigh circumference is determined by thigh measurement before harvesting an ALT flap in relation to the harvested skin island dimensions, which are described as ratios of 14% [20] to 16% [15]. In contrast, in this study, the flap ratio to thigh circumference values was determined only during the follow-up investigations and were calculated from the non-operated thigh. Nevertheless, the flap-to-thigh circumference ratio values of 16% obtained in our study is in agreement with the values in the literature. This illustrates that the use of the contralateral non-operated upper thigh, taking into account certain limitations, such as the normal anatomical variance in shape and the discrepancy in femoral circumference between the game leg and the stance leg, can nevertheless generate a sufficiently accurate control group. Altogether, these circumstances demonstrate that the thighs used in this study are consistent with the common values of operated and unoperated thighs in the literature, thus providing a coherent patient population for the evaluation of a novel three-dimensional imaging technique.

In modern medicine, assessment of surface anatomy and shape changes using three-dimensional imaging is an important component in prognostics [28] and treatment planning [1,32] for preoperative visualization of potential operative results as well as to evaluate the outcome after surgery [26,34]. It is an established method to evaluate the simulated or actual surface changes in terms of the generated distance between superimposed three-dimensional surfaces [26,34]. According to the described method in this manuscript, a desktop-based three-dimensional imaging system was used for the scans of each of the 64 patients enrolled in this study. In the control group, we were able to demonstrate that the assessment used was a valid and consistent method. As enrolled in the study, the control cohort consisted first of 34 participants without surgery on the thighs. In this group, for an average leg volume ranging from 3.7 ± 1 L of the right thigh to 3.8 ± 0.9 L of the left thigh, no statistical differences between the right and left thighs were obtained. Etzel et al. described an average total leg volume of 7.4 L by anterior and posterior digital volume quantification of the upper and lower legs in normal-weight subjects using three-dimensional surface imaging [25]. In another clinical study, the volume of the lower legs without feet was reported to be approximately 2.7 L [21], which must be subtracted from the above total leg volume to compare the volume of the investigated thighs in this study. Furthermore, anthropometric studies describe a thigh volume of 5.1 ± 0.9 L [30]. In contrast to these studies, only the anterior volume of the thighs was analyzed in our study. Corresponding to the reported volumes, the determined values of the anterior thigh volumes of the control thighs in this study agree with the volumes in the literature. Furthermore, the 30 thighs in which an ALT flap was harvested had a significantly smaller volume of 2.7 ± 0.8 L compared to the contralateral unoperated upper leg, which is consistent with the smaller thigh circumference measurements of 40 ± 2.3 cm. Nevertheless, the following considerations and limitations of the three-dimensional surface imaging evaluation used for the determination of thigh volume must be critically considered: only the surface of the anterior part of the thigh was included in the volume determination. The limits for determining the thigh volumes were defined exclusively by the surface visualization of the anterior thighs and the posterior edge by the detection range of the six cameras of the scanner. It occasionally happened that small parts of the thigh were displayed beyond 180 degrees due to the curvature of the thigh surface. Thus, a boundary was created in the region of the widest diameter of the anterior thigh in the vertical direction. Due to the specific positioning of the boundary, these small parts were not evaluated. Nevertheless, this circumstance bears the risk of minor inaccuracies during analysis. Furthermore, since the thighs do not have cylindrical symmetry in the transverse diameter, the transfer of the anterior thigh volume to a complete femoral volume is possible only in relation.

A general limitation of all studies evaluating thigh volume after ALT surgery is that changes in thigh volume or changes in thigh circumference are not only caused by the surgery itself but also by postoperative immobility and nerve damage that can lead to atrophy postoperatively, even if the muscle is not raised for an ALT flap [37].

Despite these limitations, the values obtained in this study are still in close agreement with the femoral and thigh volumes reported in the literature [21,25,30], proving optical three-dimensional surface imaging as a promising objective and reproducible tool for volume determination of thighs with an ALT donor site. Furthermore, three-dimensional surface examinations can be used in for preoperative prediction of postoperative outcomes of donor sites and other donor extremities need to be accomplished in future studies to confirm this hypothesis.

## 5. Conclusions

Desktop scanners provide reliable and accurate images for processing three-dimensional analyses of thighs. The consistency of landmarks for standardized software-based analysis of three-dimensional reconstructions proved to be a reproducible method for the scans of non-operated and operated thighs. Likewise, the method was successfully applied to the analysis of thighs after ALT flap surgery to evaluate the donor site compared to the non-operated leg. These findings need to be verified in future studies with other transplant donor sites.

## Figures and Tables

**Table 1:** Characteristics of patients.

	Control *n* = 34	ALT *n* = 30	Total *n* = 64
Female	16	12	28
Male	18	18	36
Age (years)	66 ± 11	70 ± 19	68 ± 12
Female Age (years)	62 ± 21	69 ± 26	65 ± 14
Male Age (years)	66 ± 12	64 ± 14	69 ± 12
Female Thighs Circumference (cm)	43 ± 3.3	41 ± 2.8	42 ± 3.3
Male Thighs Circumference (cm)	45 ± 2.4	40 ± 1.9	42 ± 3.3
Heigth (m)	1.7 ± 0.1	1.7 ± 0.1	1.7 ± 0.1
Weight (kg)	76 ± 21	69 ± 16	72 ± 3.4
BMI (kg/m^2^)	25 ± 4.7	23 ± 4.4	24 ± 0.2

**Table 2:** Characteristics of thighs in patients with ALT.

	Circumference ALT Thigh (cm)	Circumference Control Thigh (cm)	Flap Size (cm^2^)	Flap Width (cm)	Flap Ratio to Thigh Circumference (%)
ALT Patients *n* = 30	40 ± 2.3	44 ± 3	73 ± 43	6.9 ± 2.2	15 ± 4.6

**Table 3:** Results 3D imaging studies. N.A. = not applicable.

*n* = 128	Volume (L)	ΔVolume (L)	Maximum Deviation (mm)
ALT Thigh (*n* = 30)	2.7 ± 0.8	0.6 ± 0.4	20 ± 2.1
Control Thigh (*n* = 30)	3.3 ± 0.9		17 ± 1.9
Left Thigh Control Group (*n* = 34)	3.8 ± 0.9	0.2 ± 0.2	N.A.
Right Thigh Control Group (*n* = 34)	3.7 ± 1		N.A.

## Data Availability

All data generated for this study are available from the corresponding authors upon reasonable request.
